# Linking Survey and Administrative Data to Measure Income, Inequality, and Mobility

**DOI:** 10.23889/ijpds.v4i1.939

**Published:** 2019-01-31

**Authors:** Carla Medalia, Bruce D Meyer, Amy B O’Hara, Derek Wu

**Affiliations:** 1 U.S. Census Bureau; 2 The University of Chicago, Harris Public Policy, 1307 East 60th Street, Chicago, IL 60637; 3 National Bureau of Economic Research; 4 American Enterprise Institute; 5 Georgetown University. Massive Data Institute, McCourt School of Public Policy, Old North, 37th and O Streets, N.W. Washington D.C. 20057

## Abstract

**Background:**

Income is one of the most important measures of well-being, but it is notoriously difficult to measure accurately. In the United States, income data are available from surveys, tax records, and government programs, but each of these sources has important strengths and major limitations when used alone.

**Objectives:**

We link multiple data sources to develop the Comprehensive Income Dataset (CID), a prototype for a restricted micro-level dataset that combines the demographic detail of survey data with the accuracy of administrative measures.

**Methods:**

By incorporating information on nearly all taxable income, tax credits, and cash and in-kind government transfers, the CID surpasses previous efforts to provide an accurate and comprehensive measure of income for the population of United States individuals, families, and households. We also evaluate the accuracy of different income sources and imputation methods.

**Conclusions:**

While still in development, we envision the CID enhancing Census Bureau surveys and statistics by investigating measurement error, improving imputation methods, and augmenting surveys with the best possible estimates of income. It can also be used for policy related research, such as forecasting and simulating changes in programs and taxes. Finally, the CID has substantial advantages over other sources to analyze numerous research topics, including poverty, inequality, mobility, and the distributional consequences of government transfers and taxes.

## Introduction

Income is one of the most used variables in social science. It is employed as both an outcome and as a key explanatory variable in analyses of poverty, inequality, employment, education, health, and other issues. In the United States, income data are available from many sources, including household surveys, tax records, and data from government programs providing transfer payments. Each source has important strengths. Surveys tend to have rich demographic information that allows for the construction of families and households, supporting analyses by race, education, and other characteristics. Tax data are viewed as being very accurate and have near universal coverage when tax forms supplied by firms and government agencies are included. Finally, administrative data from government programs provide income payment information that is not captured well or at all by these other sources.

On the other hand, each of these sources has major limitations, and none of them independently offers a comprehensive and accurate measure of income. For example, certain types of income tend to be poorly reported in surveys. More than half of private pension and cash welfare payments are typically not reported, and surveys do a poor job of measuring the income of those with very high or low income [1,2]. Tax data lack important demographic details and information for many key safety net programs such as in-kind benefits and non-taxable cash transfers, which have grown considerably in recent years. Furthermore, many tax variables are only available for those who file taxes, therefore missing the low-income population that falls below the filing threshold and non-compliant non-filers. Note that the nature of underreporting in the tax records may be different from survey underreporting – and that there is more likely to be incomplete coverage of certain income sources in the tax records, such as self-employment income. Another issue is that the unit of analysis in the tax records may not necessarily reflect the economic decision-making unit, such as a household or family. Administrative data from federal and state programs are typically only available for program participants and often have little information beyond what is relevant to administer each specific program.

To address these limitations, previous research has combined income data from multiple sources. However, these studies are often one-off exercises to improve income measurement for a small target population, or are restricted to using only a few available sources of data [3,4]. The current approach of combining data from multiple sources is inefficient and repetitive. Difficult decisions about whether to conduct direct substitution or imputation, how to handle missing data, and weighting the data to obtain a representative sample have varied across previous studies, making it challenging to compare results.

To overcome these limitations, we are developing the Comprehensive Income Dataset (CID) – a resource providing an accurate and comprehensive measure of income for all United States individuals, families, and households. The CID will include demographic and income information, as well as associated details on program participation, taxes paid, and tax credits received. The CID can be used by the Census Bureau to improve surveys and statistics produced, and can increase the quality of income and poverty estimates and reduce respondent burden. The CID can be used to evaluate policies and programs, potentially enabling the Internal Revenue Service to improve studies of tax administration. In addition, we are developing this resource with the goal of making it available to researchers, potentially through the Federal Statistical Research Data Centers, secure data enclaves located across the United States.1For more information, please see: https://www.census.gov/fsrdc To build the CID, we combine the strengths of the rich social and demographic information from survey data with numerous administrative sources of income that are typically unavailable to researchers. We clearly document the methodological decisions used to combine these sources of data so that users can understand the potential implications those decisions may have for their analyses.

### Literature and Antecedents

There is a long international literature on the components of household income, the weaknesses of relying on survey data alone, and the construction of linked datasets including registers. For example, the Canberra Group Report [5], describes how other countries measure income with a combination of survey and administrative data. Many past observers have advocated more linking of survey and administrative data to measure income. Recent examples focusing on the US include Meyer, Mok, and Sullivan [1], Ziliak [6], and National Academies of Sciences [7].

The US Census Bureau produces poverty and income distribution statistics as one of its core activities. For a long time, the Census Bureau has recognized the underreporting of income components. The first non-conceptual measurement issue mentioned in a 1993 P60 series report involves income measurement error [8]:

“What corrections should be made for underreporting? Household respondents tend to underreport some types of income and the problem can be severe…The Census Bureau expects to intensify its research in this area to obtain more current and accurate estimates of the extent of the problem and to identify methods for adjusting for underreporting.” (p. ix)

Research teams with access to restricted income sources have assessed linked income data sources. Johnson and Moore [9] link Internal Revenue Service 1040 forms to the Survey of Consumer Finances and conclude that “both data sources have strengths and weaknesses that need to be understood and carefully considered before attempting to use them to answer any set of research questions” (p. 906). Davies and Fisher [10] describe the history of matching the household surveys, the Current Population Survey (CPS) and Survey of Income and Program Participation (SIPP), to Social Security Administration (SSA) earnings and benefit payments. Other researchers have sought private sector income sources. Moving beyond government administrative data, Cajner et al. [11] use Automatic Data Processing (ADP) private sector payroll data to produce new measures. Other researchers have partnered with the JPMorgan Chase Institute2For more information, see: https://www.jpmorganchase.com/corporate/institute/institute.htm to analyze income and spending using anonymized individual credit and bank account records [12,13].

These household income studies build upon years of research using administrative data and linked data to measure business activity. The Census Bureau constructed the Business Register by combining administrative data with information collected from businesses [14]. This history demonstrates that comprehensive datasets using tax data are nothing new for the Census Bureau. Many other countries also provide precedent for building business registers. The European Business Statistics Manual provides a how-to guide for business registers, including standards and guidelines. The Longitudinal Business Database (LBD) is another application of linked survey and administrative data. The LBD is a census of business establishments and firms in the United States with paid employees. It is comprised of survey and administrative records, covering all industries and all states [15].

The CID transfers these methods to household income, strengthening population-level administrative data with survey data. This is a step in the direction of income registers used in other countries. Epland [16] details the construction of Norway’s income register and explains that household surveys collect income data from the income register. Baadsgaard and Quitzau [17] describe household income and transfers in the Danish register.

As we construct the CID in the United States, we need to document the quality of the input files and of the linked dataset. The literature includes a variety of approaches to assess data quality. Oberski et al. [18] simultaneously estimate error in survey and administrative data, demonstrating their approach on linked German income data. Harron et al. [19] describe the challenges of linking administrative data, including linkage error. Schnetzer et al. [20] present a framework to assess imputations in register data, demonstrating their method on a population register with internationally used quality dimensions.

### Defining Income

To produce the CID, we need to define the measures of income that we hope to closely approximate. Discussions of income definitions often start from the Haig-Simons definition of income, which is consumption plus the change in net wealth. This measure is a reasonable goal, but not specific enough given conceptual issues, and provides limited guidance when data limitations prevent its full implementation. Unfortunately, in formulating a statistical definition of income, “there is a conflict between the goal of accurate measurement of a well-defined concept, and the desire for a measure which corresponds at least imperfectly to what seems socially relevant” [21]. Our approach will follow Ellwood and Summers [21], with some important deviations driven partly by the greater availability of data now than when they were writing 30 years ago.

We reject the idea that we should try to measure overall well-being, as that would put almost no limit on what should be included in income. Thus, we will not try to quantify the value of public schools or national defense, for example. We instead will focus on material well-being.

We include non-cash benefits if they provide for immediate material consumption or if they are fungible, freeing up resources for material consumption. In-kind benefits should capture recipient value, the amount of cash a recipient would be willing to accept in place of the noncash benefit.

We will include the full face value of in-kind transfers from the Supplemental Nutrition Assistance Program (SNAP, formerly known as food stamps) and the Special Supplemental Nutrition Program for Women, Infants, and Children (WIC), because many studies have shown that the vast majority of households consume more food than these programs provide and households tend to value them at close to face value [22]. We will include the market value of public and subsidized housing benefits, which is aided by most assistance being in the form of vouchers to purchase market housing. We will also account for state and federal income and payroll taxes. If government benefits are lowered, or they are taxed, these two actions have an equivalent effect on income and should both be recognized. As the CID develops, we will address the treatment of medical care, capital gains and pensions.

There is not a single best income measure for all purposes and disagreements over methods to employ, so we provide multiple measures. Two principles that are important in determining what types of income should be included in our measure are consistency and tractability. As Ellwood and Summers [21] put it “Consistency considerations require that the income distribution should depend on the economic substance of transactions of households not on their form.” And “There is little point in calling for the inclusion in income of benefits that cannot be measured.” (p. 12)

### Uses of the Dataset

We are currently developing a prototype of the CID, and anticipate many uses of a production version of the CID if the prototype proves successful. In this paper, we focus on three main uses of the CID: the improvement of surveys and statistical products by the Census Bureau, policy analysis and the evaluation of programs, and research.

The first use is the power of the CID to improve Census Bureau surveys and statistics. Prior research has suggested that the majority of bias in surveys is attributable to measurement error [1,23]. The CID can provide additional information on the extent of measurement error and the nature of the survey bias (for example, which demographic and family characteristics are associated with larger measurement error). Another benefit is the improvement in imputation methods to fill in missing values in surveys (in particular, item imputation). Most US imputation methods that currently account for missing values or under-reporting rely solely on other observable survey characteristics and seldom substitute administrative values or even employ them in validation checks [24,25,26,27,28].3The Census Bureau’s SIPP recently implemented administrative data in the model-based imputation of topic flags for missing values for income [25]. The CID could potentially be used to substitute administrative values for survey responses, thus reducing respondent burden, an important goal in the setting of reduced survey participation.4A 2016 workshop, which investigated respondent burden in the ACS, highlighted the importance of administrative records to reduce burden and improve data quality [29]. In addition to being good business sense, the Census Bureau is instructed to acquire and use information available from other sources instead of conducting direct inquiries (13 United States Code Section 6). Finally, the CID could be linked to other surveys to augment them with the best possible estimates of income or to create enhanced survey products that incorporate administrative data.

Second, when used for statistical purposes, the CID can be used to evaluate policies and programs, such as use by policymakers to forecast and simulate changes in programs and taxes. Many organizations - including the Office of Tax Analysis at the United States Department of the Treasury, the Joint Tax Committee, the Congressional Budget Office, and others - estimate or simulate the distributional, revenue, or incentive effects of taxes and tax reforms, often relying on tax data alone [28, 30, 31]. Linking tax data to surveys would facilitate these efforts as survey data are needed to accurately construct families and obtain demographic characteristics such as age, education, and race. These characteristics of family members are also required for microsimulations and actuarial models. For example, the incomes of family members who may not be in a given tax unit likely affect the incentives to work and to potentially form a larger tax unit. There is a long literature on the effects of one family member’s income on the labor supply of others (see Cullen and Gruber [32] for an example in the added-worker effect literature), as well as on the earnings of current or potential spouses (including cohabitants) on marriage decisions (Alm and Whittington [33] for example). The family is the natural unit for distributional analyses, as family members likely share income and plan joint expenditures in a way that unrelated roommates usually do not. The linked survey and tax data also would greatly improve the ability of analysts to characterize non-filers and incorporate them in distributional analyses.

Finally, the CID would be a valuable tool for program evaluation and research. It will be especially valuable to researchers interested in income, poverty, inequality, and mobility. Researchers could also use the CID to examine the effects of social insurance and means-tested transfer programs on poverty and the income distribution, and on other outcomes like labor force participation, health, and educational attainment.

An important extension of the CID would link population-level administrative data from several sources to Decennial Census and American Community Survey (ACS) files. This restricted microdata file with near-universal income and demographic data can be used for numerous research topics. For example, we envision the CID becoming a key resource to study inequality and poverty. By linking the CID to survey and census data at scale, one can study topics such as mobility, educational attainment, health insurance, and labor market outcomes more accurately.

## Methods

### Data

We construct the CID by linking information across household surveys and administrative tax and program participation data, all of which are available through restricted access at the United States Census Bureau. Data are linked at the individual level using persistent individual linkage keys (Protected Identification Keys or PIKs) that are appended by the Census Bureau.

### Household Surveys

To construct the CID, we focus on the following four household surveys: the CPS, the ACS, the SIPP and the Consumer Expenditure (CE) Survey, all collected by the Census Bureau, though the CPS and CE Survey are done under contract for the Bureau of Labor Statistics.

The CPS Annual Social and Economic Supplement is an annual survey of about 98,000 addresses and includes detailed questions regarding income received from numerous sources for the previous calendar year, including earned income from jobs, investment income, retirement income, and government transfers. These detailed income measures for all members of a household provide the basis for the official poverty estimates of the United States. Interviews for the CPS Annual Social and Economic Supplement are conducted from February through April each year, either in person or by phone. To begin this project, we use CPS data from reference year 2000 forward.

The ACS is a survey of about 3.5 million addresses annually that collects social, demographic, and housing information. The ACS is fielded continuously from January to December each year, and interviews are either self-administered (conducted by paper or on the internet) or interviewer-administered (in person and by phone). To begin this analysis, we use ACS data from reference year 2006 and beyond.

The SIPP is a longitudinal survey that covers approximately 50,000 households and collects detailed information at the monthly level about many different types of income and government transfers received. Earlier panels range from 2.5 to 4 years. Individuals and households were traditionally interviewed in four-month waves, but interviews are now conducted on an annual basis starting with the 2014 panel. In addition to core questions that are asked every wave, the SIPP also includes topical modules periodically that cover topics like health care, wealth, and disability. To begin this project, we use SIPP data from reference year 2001 forward.

The CE survey is the only federal household survey to collect consumption data in the United States, including household expenditures, durable good ownership, income, and demographic characteristics. The US Census Bureau collects the CE data for the Bureau of Labor Statistics via two surveys: the Interview Survey (which covers major purchases and recurring items) and the Diary Survey (which collects information about more minor or frequently purchased items). To begin this project, we use CE Interview Survey data from reference year 2000 forward.

### Tax Data

We link individuals from these surveys to a limited set of tax return data authorized under current regulations that include individual income tax returns (Form 1040), wage and tax statements (Form W-2), and distributions from pensions, annuities, and retirement plans (Form 1099-R). In addition to these data from the IRS, we use the Detailed Earnings Record (DER) file from SSA. The tax data do not generally cover non-taxable sources of income such as child support payments, veterans’ benefits, welfare benefits, supplemental security income, and gifts, bequests and inheritances. For many of these sources, we turn to administrative program data (as described in the next subsection). However, the administrative tax and program data may still tend to miss portions of certain income sources, such as business income from sole proprietors [34].

### Program Participation Data

The CID also integrates data from key government programs using both federal and state administrative records. For example, we use information on monthly receipt and amounts of Old Age Survivors and Disability Insurance (OASDI) from SSA’s Payment History Update System (PHUS) and Master Beneficiary Record (MBR). We also obtain data on monthly federally-administered Supplemental Security Income (SSI) benefits for aged, blind, and disabled people who have little or no income from the Supplemental Security Record (SSR). OASDI and SSI data are currently available at Census for the 1991-2013 CPS extracts and 1984-2008 SIPP panel extracts.

We link federal housing assistance data from the Department of Housing and Urban Development (HUD) (including the Public and Indian Housing Information Center (PIC) and Tenant Rental Assistance Certification System (TRACS) files) for calendar years 2000-2016, which cover most rental assistance benefits. These include public housing programs as well as Section 8 tenant-based vouchers and project-based assistance. However, HUD programs cover about only 70% of all subsidized rental units [35], with the rest provided by the Department of Agriculture, states, and localities - data which we do not currently have access to.

Data on enrollment in US (non-universal) government health care programs, Medicare and Medicaid, are available through the Centers for Medicare and Medicaid Services’ Medicare Enrollment Database (EDB) and Medicaid and CHIP Statistical Information System (MSIS), respectively. These monthly data span fiscal years 2000-2016 and cover nearly every state (with some states missing in more recent years). We also have monthly data on Temporary Assistance to Needy Families (TANF) from the Department of Health and Human Services (HHS), which HHS initially collects from various state agencies. States have the option to submit either sample or universe data to HHS, and 30 states submitted universe data. These data span fiscal years 2000-2014. Benefits data from the Department of Veterans Affairs (VA) are in the process of being integrated because most benefits paid by the VA are not taxable and therefore absent from the tax data, and these benefits do not appear to be accurately reported in the survey data. Longitudinal analysis will be possible using the federal data sources available back until the late 1990s.

While the aforementioned program data mostly come from federal agencies, the CID also links administrative program participation data from some states. These include data on the SNAP, which are currently available for 18 states. 5There is no common year across the eighteen states, since Mississippi only recently signed on and has delivered 2017. Sixteen states include data for 2013, and twelve states have data for the period 2013-2015. The longest panels are 2004-2016 for Indiana, 2004-2016 for Tennessee, and 2006-2018 for New Jersey.
Data on the WIC are available for nine states. 6For WIC, nine states have shared data and all nine include 2014. Six states have WIC data for 2014-2016. The longest panel of WIC data is in Washington for 2004-2016.
Public Assistance, which includes TANF data and other cash assistance, are available for 13 states. 7Thirteen states have shared Public Assistance data. Eleven of those states include 2015, and ten states have data for the period 2013-2015. The longest panels are 2004-2016 in Indiana, North Dakota, and Tennessee, and 2006-2018 in New Jersey.
Finally, we also use data from the Low Income Home Energy Assistance Program (LIHEAP), available for only one state at this time. 8Colorado has also shared LIHEAP data for 2009-14.
While it will not be possible to link these sources initially for all states, we aim to access data from enough states to allow extrapolation to the entire United States. Expanded access to data from state programs that pay benefits using federal funds may require legislative change. If and when those data become available, they will be incorporated into the CID.

### Future Sources of Data

Income concepts in the CID will expand greatly once additional tax data are received. A contract is in place to conduct joint research with the IRS statistical unit to improve income in household surveys. The Census Bureau will acquire many additional fields from Form 1040 and income amounts from Information Returns that payers submit (employers and financial institutions) including W-2, 1098, and various 1099 forms (which provide critical information for individuals who do not file an income tax return). Ultimately, we plan on having IRS data on earnings and asset income, tax liabilities and tax credits (including the Earned Income Tax Credit and the Child Tax Credit), and income from certain programs like Unemployment Insurance (UI) and Social Security (which are available on the 1099-G and 1099-SSA returns, respectively).

While the program participation data mentioned above are currently available to use in our research, we hope to expand this work using additional administrative data sources that are not yet available but could provide insight into the survey data. We continue to identify potential data sources to improve measures of earned and unearned income, particularly those absent from tax data.

For example, we are currently pursuing the acquisition of Workers’ Compensation data from several states. We plan to work with the Workers’ Compensation Research Institute to formulate appropriate strategies, such as starting with two states, Ohio and Washington, where the state itself is the only insurer and it is therefore less complicated to obtain indemnity benefits received.

Census is also exploring income measurement issues for the sharing economy. Income from platform or gig work is difficult to capture in information returns, prompting survey questions and discussions with companies in this sector.

### Analytical Plan

We plan on linking all income data in the administrative sources to the surveys while maintaining the specificity of the administrative data. This plan allows researchers to conduct analyses of income sources at a granular level while giving them the flexibility to construct broader measures of income from particular categories. Each of the survey, tax, and program datasets contains unique individual identifiers in the form of PIKs, which are anonymized versions of Social Security Numbers. PIKs are assigned by the Census Bureau using probabilistic matching methods [36]. Having access to PIKs simplifies the data linkage process considerably by circumventing the need to reconcile disparate internal identifiers across various agencies while preserving privacy.

In much of the administrative tax and program data, nearly all individuals (about 99%) are associated with a PIK. However, a smaller share of survey respondents and households is associated with a PIK (90-97%), though this share has increased in more recent survey years. To account for the inability to link all individuals or households in the surveys to a PIK, we utilize inverse probability weighting, in which we first estimate probabilities of being PIKed based on a rich set of observable characteristics and then adjust the survey weights by multiplying them by the inverse of these probabilities [37]. As a result, under certain conditions, the sample of linked survey recipients with our adjusted weights would be as representative of the population as the original full sample of survey recipients prior to linking on PIK.

When combining sources of data that were collected for different purposes, there are a variety of issues pertaining to the definition of the unit of analysis. For example, tax units may differ from household survey rosters [38,39], which may in turn differ from family units that receive program transfers. Another related issue pertains to the differing definitions of a child or dependent across the various sources of data.

In general, we associate all benefit amounts from administrative cases to survey households. When an administrative case spans multiple survey households, we distribute dollars from the administrative case to each survey household in proportion to the number of individuals linked from that case to each household. An alternative is to distribute dollars in proportion to the number of non-dependents (e.g., primary and secondary filers in tax units) linked from a case to each household. In other situations, a member of a household may be present in the administrative data source but missing from the survey data, even though the household is in the survey data. We will likely need to examine the sensitivity of results to alternative strategies to address such cases.

Since the administrative data on their own sometimes incompletely cover certain sources of income in the survey data, combining survey and administrative data yields the most complete and accurate estimates of income. For example, surveys often ask about all housing assistance, SSI, and Public Assistance - while the administrative data may only have information about housing assistance from HUD programs, federally-administered SSI, and TANF. One way to address this “gap” in the administrative data is to classify individuals as recipients of a program if they report receipt in either the survey or administrative data (probably using non-imputed observations in the survey). On one hand, this method may overestimate program receipt if we treat observations that are actual false positives as true recipients. However, we may still end up underestimating program receipt given the substantial false negatives associated with programs like non-TANF Public Assistance and non-HUD housing assistance that cannot be identified in surveys.

There are also situations where the administrative data cannot be assumed to be the true values of income. By themselves, the administrative data may miss income sources like commissions, bonuses, and tips, as well as net income after business expenses in sole proprietorships, and net rental income, the income of estates and trusts, and royalties. In all of these cases, combining the survey and administrative sources allows us to construct more accurate estimates of income than one could otherwise obtain using a particular source by itself (see, for example, Abowd and Stinson [40]).

## Results

We have begun the work of cleaning and linking the data that are currently available, and have developed initial models to analyze the data.

We plan on initially concentrating our analysis on several important issues. These include, but are not limited to: (1) Investigating the extent of survey errors including coverage error, unit and item nonresponse, and misreporting on surveys by employing “true” administrative values for the population, (2) Examining how the income distribution, poverty, inequality, and the effects of government programs change when measured using combined survey and administrative data (as opposed to survey data alone), (3) Examining changes in income measures over time for individuals as well as the population, and (4) Understanding the relationship between income, consumption, and wealth.

One of the first papers from our CID prototype development work uses the SIPP to study the effect of government programs, including Social Security, SSI, SNAP, public assistance and the earned income tax credit, on poverty reduction [41]. This paper finds that the SIPP survey data alone underestimate the poverty reduction of Social Security, public assistance and SNAP, while overestimating the poverty reduction due to SSI. The research underscores the importance of combining survey and administrative data to evaluate the efficacy of these programs.

## Conclusion

This paper describes how we are combining data from household surveys, tax records, and administrative data from federal and state government programs to develop the prototype for the Comprehensive Income Dataset. If this prototype is successful, we expect that the CID will have many important uses, including improving the Census Bureau’s household surveys, becoming a critical resource for policymakers to evaluate policies, programs and taxes, and offering better evidence for researchers investigating a diverse range of topics. The CID surpasses previous efforts to develop a source of data to analyze poverty, inequality, mobility, and the distributional consequences of government transfers, taxes, and tax credits. Because the dataset also includes details of program and tax credit receipt, the CID will be an ideal platform to simulate possible changes in welfare, social insurance programs, and tax credits, as many government agencies currently do.

## References

[1] Meyer BD, Mok WKC, Sullivan JX. Household Surveys in Crisis. Journal of Economic Perspectives. 2015; 29(4):199-226. 10.1257/jep.29.4.199

[2] Bee CA, Mitchell JW. Do Older Americans Have More Income Than We Think? [Internet]. Bureau of the Census (US); 7 2017. SESHD-WP2017-39. Available from: https://www.census.gov/content/dam/Census/library/working-papers/2017/demo/SEHSD-WP2017-39.pdf

[3] Nicholas J, Wiseman M. Elderly Poverty and Supplemental Security Income, 2002-2005. Social Security Bulletin. 2010;70(2). Available at: https://www.ssa.gov/policy/docs/ssb/v70n2/v70n2p1.html. 20560301

[4] Meyer BD, Mittag N. Using Linked Survey and Administrative Data to Better Measure Income: Implications for Poverty, Program Effectiveness and Holes in the Safety Net. 10 2015; NBER Working Paper 21676. 10.3386/w21676

[5] United Nations Economic Commission for Europe. Canberra Group Handbook on Household Income Statistics. 2011. https://www.unece.org/fileadmin/DAM/stats/groups/cgh/Canbera_Handbook_2011_WEB.pdf

[6] Ziliak J. Income, program participation, poverty, and financial vulnerability: Research and data needs. Journal of Economic and Social Measurement. 2015; 40(1-4):27-68. 10.3233/JEM-150397

[7] National Academies of Science, Engineering, and Medicine. Innovations in Federal Statistics: Combining Data Sources While Protecting Privacy. Washington (DC). The National Academies Press; 2017. 10.17226/24652 28426187

[8] U.S. Census Bureau. Measuring the Effect of Benefits and Taxes on Income and Poverty: 1992. Current Population Reports, Series P60-186RD. Suitland (MD) Department of Commerce (US), Bureau of the Census; 1993. Available at: https://www2.census.gov/library/publications/1993/demographics/p60-186rd.pdf

[9] Johnson B, Moore K. Consider the Source: Differences in Estimates of Income and Wealth From Survey and Tax Data. In: Compendium of Federal Estate Tax and Personal Wealth Studies. Volume II, Chapter 9: Comparing Administrative and Survey Data. Internal Revenue Service, Statistics of Income (US). 2012. p. 875-897. Available at: http://www.irs.gov/pub/irs-soi/11pwcompench9.pdf

[10] Davies PS, Fisher TL. Measurement Issues Associated with Using Survey Data Matched with Administrative Data from the Social Security Administration. [Internet]. Social Security Bulletin. 2009;69(2):1-12. Available at: https://www.ssa.gov/policy/docs/ssb/v69n2/v69n2p1.html 19697503

[11] Cajner T, Crane L, Decker R, Hamins-Puertolas A, Kurz C, Radler T. Using Payroll Processor Microdata to Measure Aggregate Labor Market Activity. Washington (DC): Board of Governors of the Federal Reserve System; 2018. Finance and Economics Discussion Series 2018-005. 10.17016/FEDS.2018.005

[12] Ganong P, Noel P. Consumer Spending During Unemployment: Positive and Normative Implications. [Internet]. Washington Center for Equitable Growth; Washington (DC); 4 2017a Working Paper Series. Available at: http://cdn.equitablegrowth.org/wp-content/uploads/2017/04/24150347/04252017-WP-consumer-spending-during-unemployment.pdf

[13] Ganong P, Noel P. The Effect of Debt on Default and Consumption: Evidence from Housing Policy in the Great Recession. Working Paper; 1 2017b. Available at: http://www.haas.berkeley.edu/groups/marketing/seminars/Papers/Fall2017/ganong_noel_housing_draft_2016-11-09.pdf

[14] Walker E. The Census Bureau’s Business Register: Basic Features and Quality Issues. Joint Statistical Meetings, Anaheim, CA. 1997.

[15] Jarmin R, Miranda J. The Longitudinal Business Database. 7 2002; Suitland (MD), Department of Commerce (US), Bureau of the Census: Center for Economic Studies Working Paper: 2002-17. Available at: https://www.census.gov/ces/pdf/CES-WP-02-17.pdf.

[16] Epland J. Towards a Register-based Income Statistics: The Construction of the Norwegian Income Register. Statistics Norway, Department of Industry Statistics; 2 1998. Available at: https://www.ssb.no/a/histstat/doc/doc_199805.pdf

[17] Baadsgaard M, Quitzau J. Danish Registers on Personal Income and Transfer Payments. Scandinavian Journal of Public Health. 7 2011;39(Suppl 7):103-105. 10.1177/1403494811405098 21775365

[18] Oberski DL, Krichner A, Eckman S, Kreuter F. Evaluating the Quality of Survey and Administrative Data with Generalized Multitrait-Multimethod Models. Journal of the American Statistical Association. 2017;112(520):1477-1489. http://10.1080/01621459.2017.1302338

[19] Harron K, Dibben C, Boyd J, Hjern A, Azimaee M, Barreto M, Goldstein H. Challenges in administrative data linkage for research. Big Data and Society. 12 2017;4(2):1-12. 10.1177/2053951717745678 PMC6187070 30381794

[20] Schnetzer M, Astleithner F, Cetovic P, Humer S, Lenk M, Moser M. Quality Assessment of Imputations in Administrative Data. Journal of Official Statistics. 2015;31(2):231-247. 10.1515/JOS-2015-0015

[21] Ellwood DT, Summers LH. Measuring Income: What Kind Should be In? Proceedings Vol. 1 of the Conference on the Measurement of Noncash Benefits; 12-14 12 1985; Williamsburg (VA). Bureau of the Census (US). Available at: https://www.census.gov/content/dam/Census/library/working-papers/1985/demo/measurementconf.pdf

[22] Ben-Shalom Y, Moffitt R, Scholz JK. An Assessment of the Effectiveness of Anti-Poverty Programs in the United States. In P.N. Jefferson (Ed.), The Oxford Handbook of the Economics of Poverty. 2012 Oxford: Oxford University Press.

[23] Meyer BD, Mittag N. An Empirical Total Survey Error Decomposition Using Data Combination. 10 2018; Working Paper, University of Chicago.

[24] Hokayem C, Raghunathan T, Rothbaum J. Sequential Regression Multivariate Imputation in the Current Population Survey Annual Social and Economic Supplement. Proceedings of the Joint Statistical Meeting; 8-13 8 2015; Seattle (WA). Available at: https://ww2.amstat.org/sections/srms/Proceedings/y2015/files/233986.pdf.

[25] U.S. Census Bureau. Survey of Income and Program Participation: 2014 Panel Users’ Guide. Suitland (MD) Department of Commerce (US), Bureau of the Census; 2016. Available at: https://www.census.gov/content/dam/Census/programs-surveys/sipp/methodology/2014-SIPP-Panel-Users-Guide.pdf

[26] U.S. Census Bureau. ASEC Technical Documentation 2013. Suitland (MD) Department of Commerce (US), Bureau of the Census; 2014. Available at: https://www.census.gov/prod/techdoc/cps/cpsmar13.pdf

[27] Scholz JK, Moffitt R, Cowan B. Trends in Income Support. In: Cancian M, Danziger S, editors. Changing Poverty, Changing Policies. Washington (DC): Russell Sage Foundation; 2009. p. 203-241. Available at: https://www.ssc.wisc.edu/~gwallace/Papers/Scholz%20et%20al.%20(2009).pdf

[28] Congressional Budget Office. The Distribution of Household Income and Federal Taxes, 2013. Congress of the United States Congressional Budget Office (US); 6 2016. Publication 51361. Available from: https://www.cbo.gov/sites/default/files/114th-congress-2015-2016/reports/51361-householdincomefedtaxes.pdf

[29] National Academies of Science, Engineering, and Medicine. Reducing Response Burden in the American Community Survey: Proceedings of a Workshop. Washington (DC). The National Academies Press; 2016. 10.17226/23639

[30] Joint Committee on Taxation. Estimates of Federal Tax Expenditures for Fiscal Years 2016-2020. Congress of the United States The Joint Committee on Taxation (US). 1 2017. Publication JCX-3-17. Available at: https://www.jct.gov/publications.html?func=download&id=4971&chk=4971&no_html=1

[31] Perese K. CBO’s New Framework for Analyzing the Effects of Means-Tested Transfers and Federal Taxes on the Distribution of Household Income. Congressional Budget Office Working Paper 2017-09; 2017. Available at: https://www.cbo.gov/system/files?file=115th-congress-2017-2018/workingpaper/53345-workingpaper.pdf

[32] Cullen J, Gruber J. Does Unemployment Insurance Crowd Out Spousal Labor Supply? Journal of Labor Economics. 7 2000;18(3):546-572. 10.1086/209969

[33] Alm J, Whittington LA. Does the income tax affect marital decisions? National Tax Journal. 12 1995;565-572. Available from: https://pdfs.semanticscholar.org/ea7a/44aa863cf17dc85e329a22f3796376e150e9.pdf 12320679

[34] Internal Revenue Service. Tax Gap Estimates for Tax Years 2008–2010. Washington (DC): Department of the Treasury (US), Internal Revenue Service; 2016. Available at: http://www.irs.gov/pub/newsroom/tax%20gap%20estimates%20for%202008%20through%202010.pdf

[35] Olsen EO. Housing Programs for Low-Income Households. In Means-Tested Transfer Programs in the United States, ed. Robert A. Moffitt, 365-442. Chicago (IL): University of Chicago Press; 2003. Available at: http://www.nber.org/chapters/c10259.pdf

[36] Wagner D, Layne M. The Person Identification Validation System (PVS): Applying the Center for Administrative Records Research and Applications’ (CARRA) Record Linkage Software. 2014. CARRA Working Paper Series #2014-01. Available at: https://www.census.gov/content/dam/Census/library/working-papers/2014/adrm/carra-wp-2014-01.pdf

[37] Wooldridge JM. Inverse Probability Weighted Estimation for General Missing Data Problems. Journal of Econometrics. 12 2007;141(2):1281-1301. 10.1016/j.jeconom.2007.02.002

[38] Jones MR, O’Hara AB. Do Doubled-Up Families Minimize Household-Level Tax Burden? National Tax Journal. 9 2016;69(3):613-640. 10.17310/ntj.2016.3.05

[39] Larrimore J, Mortenson J, Splinter D. Household Incomes in Tax Data: Using Addresses to Move from Tax Unit to Household Income Distributions. Washington: Board of Governors of the Federal Reserve System. 2017; Finance and Economics Discussion Series 2017-002. 10.17016/FEDS.2017.002

[40] Abowd J, Stinson MH. Estimating Measurement Error in Annual Job Earnings: A Comparison of Survey and Administrative Data. Review of Economics and Statistics. 12 2013;95(5):1451-1467. 10.1162/REST_a_00352

[41] Meyer BD, Wu D. The Poverty Reduction of Social Security and Means-Tested Transfers. ILR Review. 2018; 71(5):1106-1153. 10.1177/0019793918790220

